# Infrared Monocular Depth Estimation Based on Radiation Field Gradient Guidance and Semantic Priors in HSV Space

**DOI:** 10.3390/s25134022

**Published:** 2025-06-27

**Authors:** Rihua Hao, Chao Xu, Chonghao Zhong

**Affiliations:** Key Laboratory of Photoelectronic Imaging Technology and System, Ministry of Education of China, School of Optics and Photonics, Beijing Institute of Technology, Beijing 100081, China; 3120220549@bit.edu.cn (R.H.); 3220230681@bit.edu.cn (C.Z.)

**Keywords:** infrared image, monocular depth estimation, convolutional neural networks, supervised learning, artificial intelligence

## Abstract

Monocular depth estimation (MDE) has emerged as a powerful technique for extracting scene depth from a single image, particularly in the context of computational imaging. Conventional MDE methods based on RGB images often degrade under varying illuminations. To overcome this, an end-to-end framework is developed that leverages the illumination-invariant properties of infrared images for accurate depth estimation. Specifically, a multi-task UNet architecture was designed to perform gradient extraction, semantic segmentation, and texture reconstruction from infrared RAW images. To strengthen structural learning, a Radiation Field Gradient Guidance (RGG) module was incorporated, enabling edge-aware attention mechanisms. The gradients, semantics, and textures were mapped to the Saturation (S), Hue (H), and Value (V) channels in the HSV color space, subsequently converted into an RGB format for input into the depth estimation network. Additionally, a sky mask loss was introduced during training to mitigate the influence of ambiguous sky regions. Experimental validation on a custom infrared dataset demonstrated high accuracy, achieving a $\delta_{1}$ of 0.976. These results confirm that integrating radiation field gradient guidance and semantic priors in HSV space significantly enhances depth estimation performance for infrared imagery.

## 1. Introduction

Monocular depth estimation (MDE) has become an essential task in computational imaging, focusing on deriving depth information from a single image. Compared with active depth sensing methods such as LiDAR and structured light, which require specialized hardware and power consumption, MDE offers a passive, low-cost alternative that is especially attractive for embedded and edge computing applications. As a fundamental branch of computational imaging, MDE is crucial for various vision-based tasks, including 3D reconstruction [1], robot navigation [2], and autonomous driving [3,4]. It has attracted significant attention from both academia and industry (Intel Labs [5], Google DeepMind [6], and Toyota Research Institute [7]) due to its hardware cost-effectiveness, compact design, and ease of deployment.

Despite progress in visible-light MDE, challenges such as scene complexity, occlusion, and illumination variation continue to limit performance. Long-wave infrared (LWIR) thermal cameras, which capture emitted radiation instead of reflected light, offer distinct advantages under low-light or nighttime conditions [8,9], making them highly robust to changes in ambient lighting. However, thermal imaging also faces physically conditioned limitations: the lack of color, low texture richness, and difficulty in distinguishing objects with similar temperatures all hinder depth inference. Moreover, sky regions often exhibit extremely low thermal radiation, making it hard to extract depth cues. These factors result in poor generalization for conventional depth models when applied directly to thermal images.

To address these challenges, infrared monocular depth estimation (IR-MDE) requires a tailored approach that compensates for missing visual cues. Specifically, this study proposes the use of the HSV (Hue, Saturation, Value) color space to represent infrared images in a structured and semantically enriched manner. Unlike direct RGB-to-depth pipelines, this method constructs a meaningful multi-channel representation that enhances interpretability and robustness.

Building on this, the approach enhances infrared depth estimation by integrating semantic priors and radiation gradient guidance within the HSV representation, which is then input into a depth estimation network. This is achieved through a multi-task UNet architecture incorporating structure-aware mechanisms and specialized handling of sky regions.

The HSV color space is channel-independent [10,11]. The Value (V) channel represents intensity and brightness, the Saturation (S) channel encodes color purity or vividness, and the Hue (H) channel represents the dominant color. Compared to RGB, YCbCr, and other color spaces, this design decouples visual features in a way that is more intuitive and task-aligned.

Importantly, the method does not depend on pseudo-color mapping. Instead of assigning artificial colors to pixel intensities, object-level semantic information is incorporated into the Hue channel. This approach improves the physical interpretability of the image representation and enhances the generalization ability of the depth estimation model across various infrared scenes.

To implement the above design, this paper presents the multi-task structural aware UNet (MTSA-UNet) infrared image preprocessing framework. A Radiation Field Gradient Guidance (RGG) module is introduced to jointly guide both texture reconstruction and semantic segmentation tasks. The module improves the network’s structural detail acquisition in challenging circumstances by extracting the structural gradient from the raw infrared image and combining it with feature map gradients. The model produces a structured three-channel HSV image with semantic priors, gradience, and texture information. This structured representation serves as a high-quality, multimodal input for subsequent depth estimation.

In addition, considering that the sky regions in infrared images often lack clear radiation features and exhibit different characteristics from those in RGB images, conventional depth estimation methods tend to perform poorly in these areas. To address this, a sky region mask loss is introduced to either exclude or separately model these regions during training. This design effectively eliminates interference from invalid areas, thereby enhancing the accuracy and robustness of depth estimation.

The proposed framework opens up new possibilities for practical deployment in edge and embedded systems. In particular, since the pipeline operates directly on RAW infrared data, it is inherently compatible with the concept of *in-sensor computing*, enabling early-stage feature extraction and depth-aware processing within the imaging sensor itself.

To support and evaluate the proposed approach, a custom dataset of 2.3 k aligned infrared samples was constructed. Each sample includes 14-bit and 8-bit infrared images along with corresponding ground-truth depth map annotations. Extensive experiments and ablation studies demonstrate that the method significantly enhances the accuracy and structural consistency of infrared image depth estimation. The main contributions are summarized as follows:

- A novel HSV-based representation is introduced, where semantic priors (Hue), gradient features (Saturation), and texture information (Value) are embedded to improve the interpretability and depth estimation accuracy of infrared monocular depth estimation (MDE).
- A multi-task UNet architecture is proposed, incorporating three branches dedicated to semantic segmentation, grayscale reconstruction, and gradient feature extraction. This structure facilitates structured and complementary feature learning.
- A high-quality infrared dataset is constructed, comprising semantic segmentation labels, spatially aligned 14-bit and 8-bit infrared images, and corresponding depth maps, serving as a valuable resource for research in infrared depth estimation and scene understanding.

## 2. Related Work

### 2.1. Infrared Monocular Depth Estimation

Due to the ability of thermal imaging to provide stable image quality without relying on visible light, early research began exploring the use of thermal imaging for depth estimation. However, thermal images themselves suffer from issues such as low resolution, low contrast, and high noise, which result in poorer performance in depth estimation compared to RGB images. To address this problem, Shin et al. [12] proposed a self-supervised learning method for depth estimation based on thermal imaging, utilizing multi-spectral consistency loss (temperature and photometric consistency) to train the depth estimation network. This approach effectively enhances depth estimation accuracy in low-light and even zero-light environments. Guo et al. [13] introduced an unsupervised multi-spectral stereo depth estimation framework, combining thermal infrared and visible light images in a dual-modality approach. By imposing spatial and temporal consistency constraints, this method significantly improves depth estimation performance under all-weather conditions. It does not rely on ground-truth depth data, further enhancing the practicality of depth estimation. In subsequent work, Shin et al. [14,15] proposed another self-supervised learning method focused on monocular depth estimation using thermal imaging. They introduced an improved thermal image mapping method that enhances contrast and detail while maintaining temporal consistency, overcoming the issues of low contrast and blurry edges in thermal images. Additionally, they presented a self-supervised learning framework for thermal images, combining RGB and thermal images through feature-level adversarial adaptation to reduce modality gaps. This method, which does not require strict alignment of RGB-thermal image pairs, successfully extended self-supervised learning to cross-modal depth estimation tasks. Building on these approaches, Xu et al. [16] proposed a multi-modal fusion framework specifically designed for depth estimation in complex environments. By independently computing rough depth maps from RGB and thermal images, they introduced a novel confidence loss function to guide the depth fusion network in effectively combining the advantages of both modalities, thus improving depth estimation accuracy under nighttime and adverse weather conditions. Moreover, Li et al. [8] introduced the RIDERS (Radar-Infrared Depth Estimation for Robust Sensing) framework, which processes depth estimation through three stages: first, monocular depth estimation using thermal images, then sparse point cloud enhancement with radar data, and finally, local scale refinement of the depth. RIDERS delivers high-quality depth estimation in complex environments such as smoke and low light, surpassing the limitations of traditional methods and demonstrating its potential in autonomous driving and robotic vision. Following a different strategy, Zuo et al. [17] proposed a knowledge distillation-based method that transfers the knowledge of an RGB depth estimation model to a thermal imaging model. By leveraging the rich information from RGB images, this method improves thermal image depth estimation, particularly performing exceptionally well in the absence of labeled depth data through a confidence-aware distillation technique that predicts the depth confidence from the RGB model.

### 2.2. Infrared Image Reconstruction

Infrared 14-bit images capture a wide dynamic range, enabling detailed temperature variations across scenes. However, their high bit depth often results in low contrast and poor visibility, making it challenging to effectively visualize details. To address these issues, a variety of enhancement techniques have been proposed to improve visual quality and facilitate downstream tasks. For instance, Wang et al. [18] proposed an adaptive histogram equalization method that dynamically selects thresholds based on the image’s histogram to enhance the contrast of infrared images. Compared to traditional histogram equalization, this method avoids over-enhancing the background, making it particularly suitable for low-contrast infrared images and offering lower computational complexity. Li et al. [19] improved the double plateau histogram equalization method by introducing a normalized coefficient of variation (NCVH) as feedback, dynamically adjusting the upper and lower plateau thresholds. This algorithm shows stable performance across different scenarios, effectively enhancing image contrast while compressing the dynamic range of infrared images, making them more suitable for display devices. In another line of work, Zhang et al. [20] proposed a gradient-domain-based high dynamic range (HDR) infrared image visualization method. By balancing data constraints and gradient constraints, the method compresses the dynamic range while enhancing details. It uses histogram projection and gradient gain factor functions to preserve the global contrast of the image while effectively enhancing local details. Moreover, Bai et al. [21] introduced a multiscale new top-hat transform-based infrared image enhancement algorithm. This method extracts light and dark regions at different scales through mathematical morphology, effectively enhancing the regions of interest in the image while suppressing noise. In the context of real-time display, Durand et al. [22] presented a fast HDR image display method based on bilateral filtering. By decomposing the image into a base layer and a detail layer, the method compresses the contrast of the base layer while preserving the details. This approach effectively reduces contrast and accelerates the bilateral filtering process, providing a fast and stable solution suitable for real-time HDR image display.

Beyond traditional enhancement, several recent methods integrate perceptual modeling and task awareness. For example, Abebe et al. [23] proposed a new perceptual lightness modeling method for the dynamic range expansion and compression of high dynamic range (HDR) images. Based on the nonlinear characteristics of the human visual system, this model offers a novel approach for handling brightness perception under extreme illumination conditions, which can be integrated with existing color appearance models, ultimately contributing to more natural HDR image displays. Similarly, Gil et al. [24] introduced Fieldscale, a method focused on locality-aware rescaling, which uses a 2D field-based approach to adaptively adjust pixel gains based on local temperature variations. This method preserves image details and contrast, making it highly effective in handling thermal infrared (TIR) images with varying local temperatures. Lee et al. [25] presented TCNet, which introduces a task-adaptive tone-mapping technique. This approach dynamically adjusts the image representation for specific tasks such as object detection and depth estimation, eliminating the need for prior scene knowledge. This makes TCNet highly versatile, allowing it to be applied effectively without requiring extensive task-specific adjustments.

## 3. Method

**Figure 1:**
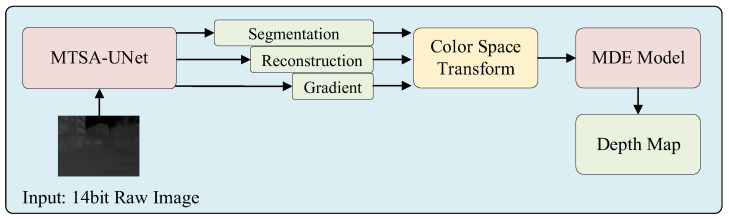
The frame diagram of the main technical strategy.

To address challenges in infrared monocular depth estimation, a two-stage framework is proposed. In the first stage, a multi-task structural aware UNet (MTSA-UNet) enhances the infrared input through three branches: texture reconstruction, semantic segmentation, and radiation gradient extraction. The outputs are fused into a structured image with three channels, where the Value channel represents intensity, the Saturation channel encodes structural gradients, and the Hue channel contains semantic priors. This enhanced representation is then used as input to the pre-trained Depth Anything V2 model for depth estimation. A sky region mask is applied during training to reduce errors in areas with weak or unreliable infrared cues. An overview of the entire pipeline is illustrated in Figure 1.

### 3.1. Choice of Color Space

This study adopts the HSV (Hue-Saturation-Value) color space to represent infrared images, due to its strong interpretability and alignment with task-specific features. Each channel in HSV carries a distinct type of information. The Value channel captures the intensity and texture of the infrared image. The Saturation channel encodes structural details guided by radiation gradients. The Hue channel integrates semantic priors from segmentation outputs.

Unlike RGB or YCbCr, where brightness and color components are entangled or lack semantic clarity, HSV decouples visual cues in a physically meaningful way. This separation allows for targeted feature representation and better control over each modality. The design ensures that texture, structure, and semantics are encoded independently but complementarily, reducing feature overlap and ambiguity.

The use of the Hue channel for semantic encoding is particularly beneficial. It supports object-level scene understanding, which improves generalization and robustness in diverse infrared environments. By structuring the image into interpretable components, the HSV space forms a strong foundation for downstream depth estimation tasks, enhancing both accuracy and model reliability.

### 3.2. MTSA-UNet Network Structure

**Figure 2:**
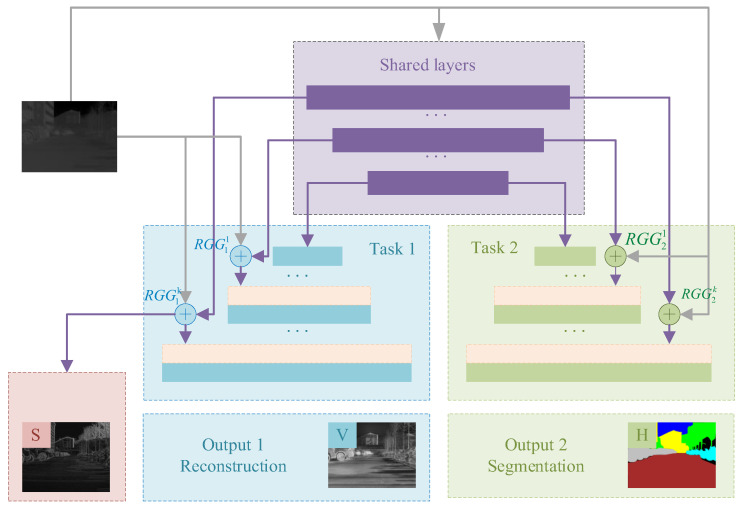
Structure of MTSA-UNet.

This section introduces the proposed MTSA-UNet (Multi-Task Structure-Aware UNet), with the overall network architecture illustrated in Figure 2. MTSA-UNet builds upon a multi-task U-Net structure, specifically designed for infrared image reconstruction and semantic understanding in complex thermal scenes. Different sub-tasks, including texture reconstruction and semantic segmentation, are trained jointly, utilizing a shared encoder while preserving distinct decoder branches for each task. To improve structural consistency, a Radiation Field Gradient Guidance (RGG) module is introduced. This module extracts structural gradients directly from the raw infrared image and fuses them with intermediate feature map gradients, guiding both decoding branches with explicit structural cues. Each decoder integrates shared encoder features and RGG-guided features, promoting task-specific refinement while preserving spatial detail.

MTSA-UNet maintains the localization precision of the original U-Net architecture while leveraging multi-task learning and structure-aware guidance to enhance performance across all outputs. Further details on the MTSA-UNet design and the RGG module are provided in Section 3.2.1 and Section 3.2.2.

#### 3.2.1. Basic Architecture of the MTSA-UNet Network

**Figure 3:**
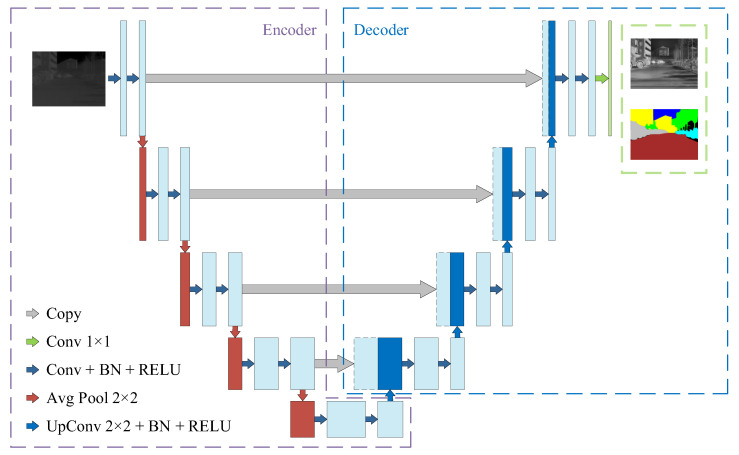
Structure of U-Net.

The MTSA-UNet adopts the classic U-Net architecture [26] as its fundamental framework. As shown in Figure 3, MTSA-UNet preserves the characteristic U-shaped structure of the original U-Net, enabling the fusion of multi-scale spatial and contextual information essential for infrared image understanding.

The encoder consists of four downsampling stages. Each stage comprises two 3 × 3 convolutional layers, followed by Batch Normalization and ReLU activation. The feature map resolution is halved using a 2 × 2 max pooling operation, and the number of channels is progressively increased from 32 to 256. After the encoder, a bottleneck layer further expands the feature dimension to 512 using similar convolutional operations.

To handle multiple tasks, MTSA-UNet includes separate decoder branches for different outputs, such as texture reconstruction and semantic segmentation. Each decoder contains four upsampling blocks, implemented using transposed convolution followed by batch normalization, ReLU, and a double convolution block. Skip connections from encoder layers are added to corresponding decoder stages to preserve spatial detail.

The reconstruction output is generated through a 1 × 1 convolution followed by a sigmoid activation, producing an infrared-normalized grayscale image. The segmentation output is generated via a 1 × 1 convolution that outputs six channels, corresponding to semantic classes. MTSA-UNet is implemented in PyTorch 2.1 and trained using the AdamDelta [27] optimizer.

#### 3.2.2. Radiation Field Gradient Guidance (RGG) Module

Inspired by attention-based MTL structures such as EEA-Net [28] and MTA-UNet [29], the RGG module is introduced to enhance structural awareness in the decoding process. The RGG module integrates structural gradient cues extracted directly from the input infrared image into the decoding process, guiding both texture reconstruction and semantic segmentation tasks.

Specifically, the RGG module computes gradient maps along horizontal and vertical directions using first-order difference operators. These gradient features are then fused with upsampled decoder features through a learnable attention mechanism to strengthen edge information. Additionally, the skip connections between the encoder and decoder are improved; instead of direct feature addition, the encoder features are first modulated by gradient-enhanced attention maps before fusion.

Given a feature map $f^{k}$ from the decoder and a gradient map ∇I generated by the Gradient Computation Module (GCM), the RGG-guided process can be expressed as:

(1) $$RGG_{m}^{k}(f^{k},I)=\alpha \cdot \sigma (Conv({\left(\nabla I\right)}^{\gamma }\cdot f^{k}))+(1-\alpha )\cdot \sigma (Conv({\left(\nabla I\right)}^{\gamma }))$$

where ${RGG}_{m}^{k}\;$ denotes the task-dependent gradient-guided attention module. To effectively integrate both structural and feature cues, the RGG module introduces a balancing coefficient α to control the fusion of feature and gradient guidance. Specifically, α  determines the weight between the modulation of task features $f^{k}$ and the structural features derived from the gradient of the input image ∇I. In this work, α is empirically set to 0.5, equally weighting both components. This choice ensures that the decoder benefits from semantic representations while still retaining the structural fidelity provided by gradient cues, which are especially important for infrared imagery with weak texture and low contrast. The setting of α=0.5 offers a practical trade-off, balancing generalization and feature alignment without introducing significant instability during training.

#### 3.2.3. Gradient Computation Module (GCM)

**Figure 4:**
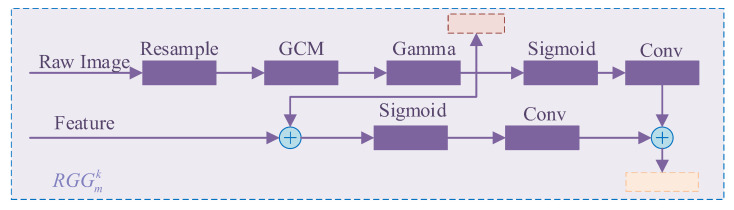
Structure of RGG.

The computation process of the Gradient Computation Module is illustrated in Figure 4 and is described as follows:

Given an input image I, the gradient magnitude map ∇I is computed along the horizontal and vertical directions using a simple first-order difference:

(2) $$\nabla_{x}=\left|I_{i,j+1}-I_{i,j}\right|,\nabla_{y}=\left|I_{i+1,j}-I_{i,j}\right|$$

Finally, the total gradient magnitude map is obtained by summing the horizontal and vertical gradients:

(3) $$\nabla I=Clamp(\nabla_{x}+\nabla_{y},\min=1\times 10^{-6})$$

### 3.3. Monocular Depth Estimation Backbone

#### 3.3.1. Selection of Depth Anything V2

At present, in the ranking of monocular depth estimation tasks on the KITTI benchmarks, the published models with better accuracy and robustness include SPIDepth [30], Metric3Dv2 [31], Depth Pro [32], Depth Anything V2 [33], etc. As of the submission of this paper, Depth Anything V2 achieves comparable accuracy to existing state-of-the-art methods and offers fast inference speed. Therefore, this work adopts it as the baseline for depth estimation. To perform monocular depth estimation on infrared images, this work adopts Depth Anything V2 as the backbone depth estimation model. Depth Anything V2 is a mature and high-performing architecture that demonstrates excellent accuracy and inference speed on standard benchmarks. Without modifying its internal structure, Depth Anything V2 is directly integrated into our pipeline as the depth estimation component.

#### 3.3.2. Color Space Transform

To ensure compatibility with the RGB-based Depth Anything V2 model, the three-channel output of MTSA-UNet is converted into an RGB-format image. The three channels respectively represent texture, semantic, and structural information, corresponding to Value, Hue, and Saturation in an HSV representation. This structured representation is then mapped to RGB space through a fixed transformation. The resulting image retains critical infrared features while meeting the input requirements of the depth estimation network, enabling accurate training and inference in the infrared domain.

#### 3.3.3. Sky Region Supervision

In conventional monocular depth estimation tasks, the sky region is often excluded or ignored due to its undefined or infinite depth. However, in infrared imagery, the sky region exhibits distinct characteristics, typically appearing as a low-temperature area with relatively stable radiation patterns. This property is leveraged to provide explicit supervision over the sky region, improving overall scene understanding and boundary consistency.

In the proposed infrared depth estimation framework, the MTSA-UNet network is employed not only for structural and semantic enhancement of infrared images but also to generate precise sky region masks via semantic segmentation. Leveraging this segmentation output, we introduce a sky-aware mask loss function in the second stage to provide explicit supervision for the sky region, thereby improving the completeness and robustness of the overall scene depth estimation.

We define the sky region using a semantic segmentation mask $M_{sky}\in {\{0,1\}\;}^{H\times W}$, where the pixels corresponding to the sky are marked as 1. The sky depth value is predicted as 99% of the maximum depth value in the training dataset, representing the furthest point in the scene.

The sky supervision loss $L_{sky}$ as follows:

(4) $$L_{\mathrm{sky}}=\frac{1}{N_{\mathrm{sky}}}\sum_{(i,j)\in M_{\mathrm{sky}}}{\left\|D_{pred}^{\left(i,j\right)}-D_{ref}^{\mathrm{sky}}\right\|}_{1}$$

where $D_{pred}^{\left(i,j\right)}\;$ is the predicted depth, $D_{ref}^{sky}$ is a reference depth, and $N_{sky}\;$ is the number of sky pixels. This ensures the sky is interpreted coherently in the thermal domain, even without precise GT depth.

### 3.4. Loss Functions

The training of the proposed framework is driven by a combination of multi-task losses, each targeting a different sub-network and objective.

(1) Image Reconstruction Loss $L_{rec}$

This term supervises the UNet’s output $I_{8bit}^{recon}$ using ground truth 8-bit images $I_{8bit}^{GT}$:

(5) $$L_{rec}={\left\|I_{8bit}^{recon}-I_{8bit}^{GT}\right\|}_{1}$$

(2) Segmentation loss $L_{seg}$

The semantic segmentation branch of MTSA-UNet is supervised using the standard Cross-Entropy (CE) loss, which quantifies the pixel-wise classification error between the predicted segmentation map and the ground truth labels.

Let $p_{i}^{\left(c\right)}\;$ denote the predicted probability of a pixel i belonging to the class c and let $y_{i}$ be the ground truth class label for a pixel i. The CE loss over all pixels in the segmentation map is computed as follows:

(6) $$L_{seg}=-(\frac{1}{N})\sum_{i=1}^{N}\sum_{c=1}^{C}\delta (y_{i}=c)\cdot log(p_{i}^{\left(c\right)})$$

where

N is the total number of pixels;
C is the number of segmentation classes;
$\delta (y_{i}=c)$ is an indicator function that equals 1 when the ground truth class of a pixel i is c, and 0 otherwise;
$log(p_{i}^{\left(c\right)}$) is the log-probability of the predicted class.
(3) Depth Estimation Loss $L_{depth}$

The scale-invariant logarithmic loss (SiLogLoss), as utilized by the model authors in [33], is applied as the depth estimation loss function within the principal deep supervision framework.

(4) Sky Supervision Loss $L_{sky}$

As described in Section 3.3.3, this term applies only to pixels identified as sky to enforce realistic shallow depth predictions.

## 4. Results

This section presents the experimental results used to evaluate the effectiveness and robustness of the proposed method. The dataset, implementation details, and evaluation metrics are first described. Subsequently, benchmark comparisons with existing preprocessing approaches are conducted, followed by ablation studies on key modules. Finally, the reconstruction and segmentation performance of MTSA-UNet is analyzed, and the model’s generalization capability is assessed under varying illumination conditions.

### 4.1. Dataset Description

**Figure 5:**
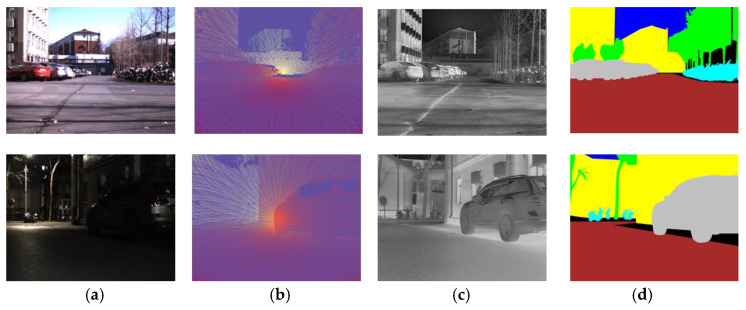
Example of dataset. (**a**) RGB Image; (**b**) depth map generated by LiDAR point projection; (**c**) infrared Image; (**d**) semantic mask.

A high-quality infrared depth estimation dataset was first constructed. The system includes an iRay LGCS121 LWIR camera, a Livox Mid-40 LiDAR, and two Alvium 1800c–240c RGB cameras. To ensure dense point coverage, the LiDAR collects data over a fixed 5s integration period, generating about 500,000 3D points per scene. Figure 5 shows an example of our dataset.

The dataset includes 2292 scenes captured in autumn, winter, and spring. It covers different illumination conditions: daytime, sunset, and nighttime. A total of 57.1% of the scenes were collected in low-light or complex illumination. Both the 14-bit infrared images and the 8-bit grayscale images processed by the iRay infrared camera built-in algorithm (hereinafter referred to as IRCBA), which includes dual-platform histogram equalization, Digital Detail Enhancement (DDE), and frequency domain filtering, were saved during the capture process.

Each scene includes RGB images, LiDAR depth maps, infrared images, and semantic masks. Six categories were annotated: sky, buildings, cars, bicycles, roads, and trees. These are common in our scenes and important for depth estimation. The semantic labels help guide learning. The categories are extendable if needed.

### 4.2. Implementation Details and Metrics

To promote stable convergence and facilitate effective multi-task learning, a two-stage training strategy is employed, separating the training of the MTSA-UNet reconstruction module from that of the Depth Anything V2 depth estimation network. This enables each sub-network to focus on its own objective. All experiments are conducted on a single NVIDIA RTX 3090 GPU with 24 GB of memory. Data augmentation is applied consistently in both stages to improve model generalization. Techniques include random center cropping, brightness jittering, and horizontal flipping to simulate diverse thermal imaging conditions. Specifically, the following:

Random center cropping: A crop size ratio is randomly sampled from the range [0.8, 1.0] relative to the original resolution and applied with a probability of 0.1.

Brightness jittering: Brightness adjustment is performed using a factor randomly selected from [0.8, 1.2], applied with a probability of 0.1.

Horizontal flipping: The image is flipped horizontally with a probability of 0.5.

#### 4.2.1. Stage I: Pre-Training of MTSA-UNet

Due to the high resolution of the input infrared images (1024 × 768), training with large batch sizes is limited by GPU memory. To address this, gradient accumulation over four steps is used to simulate a larger batch size during training.

Stage I consists of two steps. In Step I-1, MTSA-UNet is trained on the full dataset without semantic labels. The goal is to learn texture reconstruction and structural gradients from the raw 14-bit infrared images. The model reconstructs 8-bit grayscale images and extracts gradient features, supervised by a reconstruction loss. The learning rate is set to $1\times 10^{-4}$, and the model is trained for 50 epochs.

In Step I-2, the semantic segmentation task is fine-tuned using 800 labeled infrared images. Rather than freezing the encoder and auxiliary branches, a soft multi-task learning approach is applied, keeping all branches active while adjusting their loss contributions. The segmentation task is weighted at 1.0, and the reconstruction task at 0.1. Learning rates are set to $1\times 10^{-4}$ for the segmentation branch and $1\times 10^{-5}$ for the encoder and the texture reconstruction. The model is trained for 50 epochs.

The segmentation branch is optimized using $L_{seg}$ This design encourages feature sharing while allowing the semantic branch to benefit from structural priors learned during pretraining. The 800 labeled images are randomly divided into 600 for training, 100 for validation, and 100 for testing to ensure a balanced distribution.

#### 4.2.2. Stage II: Joint Training with Depth Anything V2

In the second stage, Depth Anything V2 is fine-tuned for monocular depth estimation using a total of 2292 thermal infrared images with corresponding depth annotations. The dataset includes diverse scenes, divided into 1500 training images, 400 validation images, and 392 test images. Raw thermal images are first processed through the pretrained MTSA-UNet and transformed into RGB format via the color space transform module. The resulting pseudo-RGB images are used as input to Depth Anything V2. Fine-tuning is performed end-to-end using a batch size of 4 for 50 epochs, with an initial learning rate of $\;1\times 10^{-5}$. The overall training objective is formulated as

(7) $$L=L_{\mathrm{depth}\;}+\lambda_{\mathrm{sky}\;}L_{\mathrm{sky}\;}$$

where $L_{sky}$ denotes the standard depth estimation loss, $L_{sky}$ represents the auxiliary supervision loss applied to the sky region, and $\lambda_{sky}$ balances the two components. The lower weight for sky supervision reflects the prioritization of depth estimation accuracy.

Four standard evaluation metrics widely used in monocular depth estimation are adopted: Absolute Relative Error (AbsRel), Squared Relative Error (SqRel), Root Mean Square Error (RMSE), and threshold accuracy, following definitions in [34].

### 4.3. Benchmark Experiments

Under the same training dataset and conditions, three representative infrared image preprocessing methods were selected for comparative experiments: Histogram Equalization (HE), Contrast Limited Adaptive Histogram Equalization (CLAHE), and IRCBA. Table 1 presents the quantitative evaluation results of these methods on the infrared monocular depth estimation task.

**Figure 6:**
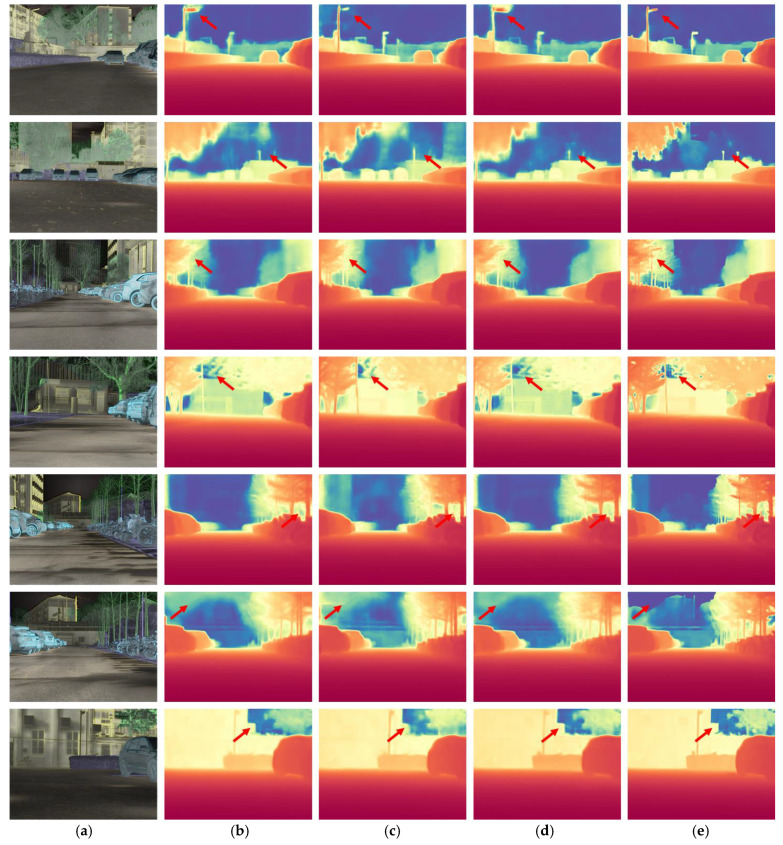
Depth estimation results on infrared images processed by different methods. (**a**) Infrared image processed by color space transform; (**b**) using HE as infrared preprocess; (**c**) using CLAHE as infrared preprocess; (**d**) using IRCBA as infrared preprocess; (**e**) our method.

As shown in Table 1, our approach outperforms all baselines across key metrics ($\delta_{1},\;\delta_{2},\;\delta_{3},\;Abs\;Rel,\;Sq\;Rel,\;and\;RMSE$), with $\delta_{1}=0.967$ and RMSE=3.705, confirming the effectiveness of our design. Furthermore, as illustrated in Figure 6, our method demonstrates superior sky-region handling after introducing the sky mask loss. The depth predictions show clear sky-background separation and well-preserved object boundaries, indicating improved semantic understanding and structural perception. The proposed method encodes infrared images into HSV space, where semantic priors, radiation gradient cues, and texture features are embedded into the H, S, and V channels, respectively. This structured representation enables the network to capture more discriminative depth cues from infrared images.

### 4.4. Advantages of the Multi-Task UNet Architecture

To evaluate the effectiveness of the proposed MTSA-UNet architecture, comparison experiments on texture reconstruction and semantic segmentation were conducted. For fairness, training protocols were aligned between single-task and multi-task models. In the texture reconstruction single-task model, only Stage I-1 (pre-training) was performed using the full dataset, without semantic labels. All other training settings matched those of the multi-task model’s Stage I-1. For the semantic segmentation single-task model, encoder weights from Stage I-1 were used for initialization, and only the segmentation branch was trained. Training settings were consistent with Stage I-2 of the multi-task model. Standard cross-entropy loss was used without any reconstruction loss. Detailed results are presented in the following subsections.

#### 4.4.1. Comparison of Texture Reconstruction Performance

**Figure 7:**
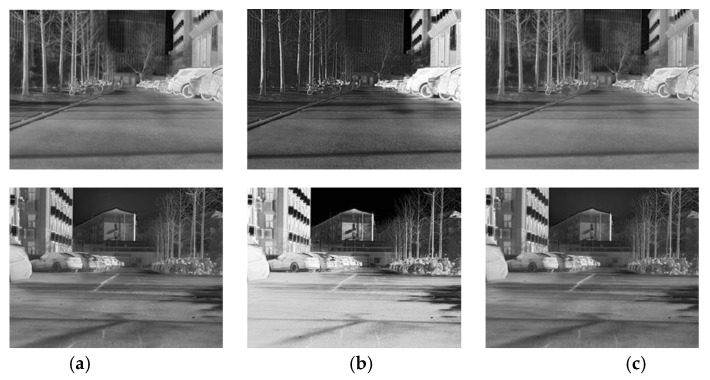
Comparison between single-task and multi-task reconstruction. (**a**) Multi-task reconstruction result. (**b**) Single-task reconstruction result. (**c**) Ground truth (IRCBA).

Table 2 presents a comparison between multi-task learning and single-task texture reconstruction in terms of image quality metrics, including SSIM, PSNR, and entropy. As shown in Figure 7, the model trained with the multi-task learning strategy consistently outperforms the single-task baseline across all evaluation metrics. Notably, the PSNR is substantially improved from 15.456 to 28.481, indicating that the multi-task design effectively enhances the reconstruction quality.

From a subjective visual assessment, the single-task grayscale reconstruction model tends to overemphasize edge features, leading to overly high contrast and the suppression or loss of fine texture details. This degrades the naturalness and fidelity of the reconstructed images. In contrast, the multi-task learning framework, by simultaneously optimizing structural edge preservation and semantic segmentation tasks, significantly alleviates this issue. The reconstructed images maintain better structural integrity while preserving finer texture details, resulting in improved perceptual quality and higher information entropy (increasing from 6.859 to 7.377).

#### 4.4.2. Comparison of Segmentation Performance

**Figure 8:**
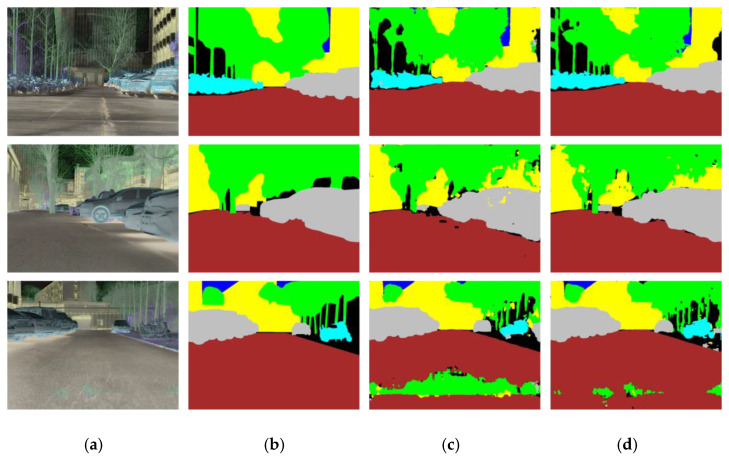
Comparison between single-task and multi-task segmentation. (**a**) Infrared HSV image; (**b**) ground truth; (**c**) single-task segmentation result; (**d**) multi-task segmentation result.

Both quantitative and qualitative results demonstrate the effectiveness of the proposed multi-task learning strategy. As shown in Table 3, the mIoU improves from 0.5092 to 0.6313 when multi-task learning is employed. Furthermore, as illustrated in Figure 8, the visual comparisons reveal that the segmentation boundaries become significantly clearer and more accurate, indicating that the multi-task framework helps the network better capture semantic structures and fine details.

### 4.5. Robustness Verification Under Diverse Illumination Conditions

**Figure 9:**
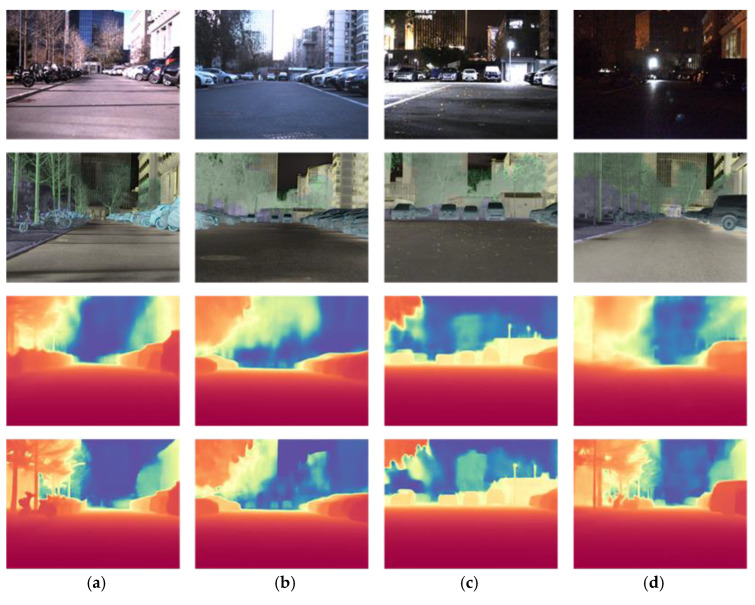
Depth estimation under different illumination conditions. From top to bottom, RGB images, infrared by HSV enhanced, RGB inference, and infrared inference. (**a**) Data collected between 09:00 and 11:00 in daylight conditions, (**b**) data acquired one hour before sunset, (**c**) data collected during nighttime between 19:00 and 20:00, and (**d**) data captured in the early morning between 02:00 and 03:00.

To further investigate the reliability of depth estimation under varying illumination, a detailed comparative analysis of RGB and infrared (IR) modalities was conducted across consistent scenes under three representative illumination conditions. As shown in Table 4 and Figure 9, the IR-based system consistently delivered robust performance, particularly under degraded visual environments.

(1) Daylight and Oblique Sunlight Performance: Under ample sunlight conditions, both the infrared (IR) and RGB-based systems demonstrated high depth estimation accuracy, with $\delta_{1}$ scores of 0.974 (IR) and 0.976 (RGB). However, the IR model achieved a lower RMSE (3.018 vs. 3.503), indicating smoother error distribution and enhanced structural coherence. In oblique sunlight scenarios such as dusk, IR significantly outperformed RGB with a $\delta_{1}$ of 0.963 versus 0.948, and also showed improved AbsRel (0.048 vs. 0.072) and RMSE (3.351 vs. 4.119). This demonstrates IR’s superior robustness to extreme illumination angles and shadow artifacts, as it relies on thermal radiation rather than ambient visible light.
(2) Low-Light (Nighttime) Resilience: In nighttime conditions, the IR system maintained relatively high accuracy ($\delta_{1}=0.946$) and structural integrity (SqRel=0.793), outperforming the RGB model ($\delta_{1}=0.909,\;SqRel=0.827$), despite both experiencing increased error. The IR model’s lower RMSE (5.126 vs. 5.266) further demonstrates its advantage in thermally differentiated scenes.

Overall, the IR-based depth estimation system exhibited superior stability and consistency across diverse illumination conditions, with $\delta_{1}$ fluctuations kept within 2.6% from day to night. These findings validate the method’s effectiveness for real-world scenarios with diverse illumination, such as autonomous navigation and robotics in unstructured environments.

### 4.6. Ablation Study on RGG Module

An ablation study was conducted to assess the RGG module. Two models were trained: one with RGG, one without. All settings remained the same, including data splits, optimizer, learning rate, batch size, epochs, and losses.

As shown in Table 5, the RGG-enhanced model achieves higher PSNR (28.48 vs. 27.09) and SSIM (0.869 vs. 0.837), indicating improved reconstruction quality, better visual consistency, and clearer structures. Information entropy also increases (7.377 vs. 7.333), approaching the ground truth level (7.399), suggesting richer texture and higher detail density in the reconstructed images.

Semantic segmentation also benefits from the RGG module, as shown in Table 6, where mIoU improves from 0.5988 to 0.6313. This indicates that structural gradient guidance enhances not only low-level features but also high-level semantic understanding. Visual results show sharper, more coherent object boundaries, confirming the module’s effectiveness in refining spatial semantics in infrared imagery.

### 4.7. Ablation Study on the Sky Region Mask Loss

**Figure 10:**
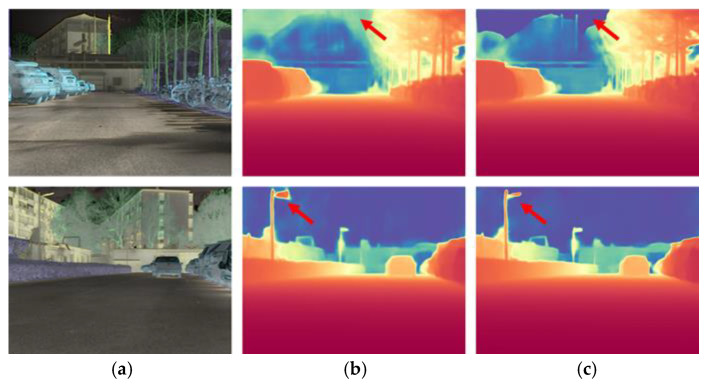
Depth estimation comparison between with and without sky mask loss. (**a**) Infrared HSV image; (**b**) training without sky mask loss; (**c**) training with sky mask loss.

To investigate the effect of the sky region mask loss, a comparative experiment was conducted by removing it during Stage II training, while keeping all other training settings, datasets, and evaluation protocols identical. Visual comparisons are provided in Figure 10, and quantitative results are listed in Table 7.

Although the introduction of the sky mask loss does not lead to a significant improvement in quantitative metrics, it brings enhancements in qualitative results. Specifically, the reconstructed depth maps exhibit finer texture details and more accurate boundary restoration, particularly in regions that were previously blurred or structurally ambiguous. The underlying reason is that the sky loss forces the model to learn additional information to better understand the sky regions, thereby enhancing its ability to extract global structural features from the image. As a result, the overall structure perception capability of the model is further strengthened, leading to clearer object contours, better preservation of fine details, and a substantial improvement in the practical performance of infrared image depth estimation.

## 5. Discussion and Conclusions

Despite the high accuracy achieved by our framework, several limitations should be acknowledged. One limitation exists in utilizing 8-bit greyscale images, produced by the IRCBA, as supervisory targets for the reconstruction task. Although these images do not capture the full radiometric detail of the infrared spectrum, they preserve essential structure and texture. This makes them well-suited for guiding perceptual reconstruction, especially in tasks where relative contrast and edge information are more critical than absolute radiometric accuracy.

Another consideration is the limited amount of labeled data for semantic segmentation. Our model is trained on only 800 annotated infrared images, which may restrict segmentation precision. However, since semantic outputs are used only to encode priors into the Hue channel, the model is not highly sensitive to segmentation accuracy. Moreover, prior studies [35] have demonstrated that neural networks in vision tasks are more responsive to luminance features than to chrominance, supporting our design choice to prioritize structural and brightness cues over precise semantic segmentation in the HSV-based depth representation.

This paper presented an end-to-end infrared monocular depth estimation framework that incorporates semantic priors and radiation field gradient guidance within an HSV-based encoding strategy. The proposed approach addresses key limitations of infrared imaging, such as texture sparsity and lack of color information, by explicitly decomposing scene information into Hue (semantic priors), Saturation (gradient structure), and Value (texture details), enabling a semantically guided and structurally aware representation for depth learning.

To achieve this, this paper designed a multi-task UNet architecture that performs grayscale reconstruction, semantic segmentation, and gradient extraction jointly. A Radiation Field Gradient Guidance (RGG) module was embedded to enhance structural representation, while a sky region mask loss was introduced to mitigate errors in texture-less sky areas. Together, these components allow the model to better leverage the unique characteristics of infrared images.

Experimental results on a custom-built infrared dataset demonstrate that the proposed framework significantly improves depth estimation accuracy and structural consistency, achieving a $\delta_{1}$ accuracy of 0.976. Notably, even with limited semantic labels, the HSV encoding framework enables effective use of semantic priors without being overly sensitive to segmentation precision.

The proposed infrared monocular depth estimation framework, with its robustness to low-light and adverse weather conditions, shows great potential in practical applications such as autonomous driving, robotic navigation, and nighttime surveillance. Its ability to operate directly on raw infrared data makes it especially suitable for deployment on embedded and edge devices.

In future work, we plan to (1) scale up the semantic segmentation module using larger annotated datasets, (2) explore domain adaptation for cross-sensor generalization, and (3) investigate lightweight implementations for embedded deployment scenarios.

## Figures and Tables

**Table 1:** Comparison of different infrared image processing methods on depth estimation performance. The best results are in bold, and the second best are underlined.

Method	$\delta_{1}$	$\delta_{2}$	$\delta_{3}$	Abs Rel	Sq Rel	RMSE
HE	0.945	0.987	**0.996**	0.096	0.555	3.940
CLAHE	0.950	0.986	0.995	0.059	0.534	3.880
IRCBA	0.958	0.984	0.993	0.061	0.549	3.768
Ours	**0.967**	**0.989**	**0.996**	**0.054**	**0.436**	**3.705**

**Table 2:** Texture reconstruction comparison between single-task and multi-task learning.

Muti-Task	SSIM	PSNR	Entropy
Yes	0.869	28.481	7.377
No	0.529	15.456	6.859

**Table 3:** Semantic segmentation comparison between single-task and multi-task learning.

Muti-Task	mIoU
Yes	0.6313
No	0.5492

**Table 4:** MDE Performance under different illumination conditions. The best results are in bold, and the second best are underlined.

Environment	$\delta_{1}$	$\delta_{2}$	$\delta_{3}$	Abs Rel	Sq Rel	RMSE
Daytime(IR)	0.974	**0.994**	**0.996**	0.041	0.422	**3.018**
Dusk(IR)	0.963	0.992	**0.996**	0.048	0.529	3.351
Night(IR)	0.946	0.983	0.993	0.095	0.793	5.126
Daytime(RGB)	**0.976**	0.992	**0.996**	**0.039**	**0.282**	3.503
Dusk(RGB)	0.948	0.987	0.995	0.072	0.494	4.119
Night(RGB)	0.909	0.976	0.993	0.086	0.827	5.266

**Table 5:** Evaluation of image reconstruction quality with and without the RGG Module.

RGG	SSIM	PSNR	Entropy
Yes	0.869	28.481	7.377
No	0.837	27.093	7.332

**Table 6:** Evaluation of semantic segmentation accuracy with and without the RGG Module.

RGG	mIoU
Yes	0.6313
No	0.5988

**Table 7:** Evaluation of MDE accuracy with and without the sky mask loss.

Sky Loss	$\delta_{1}$	$\delta_{2}$	$\delta_{3}$	Abs Rel	Sq Rel	RMSE
No	0.964	0.987	0.996	0.060	0.482	3.805
Yes	0.967	0.989	0.996	0.054	0.436	3.705

## Data Availability

The data presented in this study are not publicly available due to institutional restrictions, but may be made available from the corresponding author on reasonable request.

## Abbreviations

The following abbreviations are used in this manuscript:

MDE	Monocular depth estimation
RGG	Radiation Field Gradient Guidance
MTSA	Multi-task structural aware
LWIR	Long wave infrared
GT	Ground truth
IRCBA	Infrared camera built-in algorithm
HE	Histogram equalization
CLAHE	Contrast-limited adaptive histogram equalization
SSIM	Structural similarity index measure
PSNR	Peak signal-to-noise ratio
mIoU	Mean intersection over union
Abs Rel	Absolute Relative Error
Sq Rel	Squared Relative Error
RMSE	Root Mean Square Error
